# Reference intervals for plasma sulfate and urinary sulfate excretion in pregnancy

**DOI:** 10.1186/s12884-015-0526-z

**Published:** 2015-04-17

**Authors:** Paul Anthony Dawson, Scott Petersen, Robyn Rodwell, Phillip Johnson, Kristen Gibbons, Avis McWhinney, Francis Gerard Bowling, Harold David McIntyre

**Affiliations:** Mater Research Institute University of Queensland, TRI, Woolloongabba QLD, Brisbane, Australia; Mater Research, South Brisbane QLD, Brisbane, Australia; Mater Mothers’ Hospital, Mater Health Services, South Brisbane QLD, Brisbane, Australia; Queensland Cord Blood Bank At The Mater, Mater Health Services, South Brisbane QLD, Brisbane, Australia; Pathology Department, Mater Health Services, South Brisbane QLD, Brisbane, Australia; Mater Children’s Hospital, Mater Health Services, South Brisbane QLD, Brisbane, Australia; Mater Clinical School, University of Queensland, South Brisbane QLD, Brisbane, Australia

**Keywords:** Sulfatemia, Human gestation, Cord blood, Maternal sulfate levels

## Abstract

**Background:**

Sulfate is important for fetal growth and development. During pregnancy, the fetus relies on sulfate from the maternal circulation. We report reference intervals for maternal plasma sulfate levels and fractional excretion index (FEI) for sulfate in pregnancy, as well as sulfate levels in cord blood from term pregnancies.

**Methods:**

Plasma and urine were collected from 103 pregnant women of 10-20 weeks gestation and 106 pregnant women of 30-37 weeks gestation. Venous cord plasma was collected from 80 healthy term babies. Sulfate levels were measured by ion chromatography. Plasma and urinary creatinine levels were used to calculate FEI sulfate in pregnant women. Analyses provide reference intervals, and explored the relationship between maternal sulfate data with several prenatal factors.

**Results:**

Median maternal plasma sulfate levels were 452 μmol/L and 502 μmol/L at 10-20 and 30-37 weeks gestation, respectively, and inversely correlated with FEI sulfate median values of 0.15 and 0.11. Overall reference intervals were 305-710 and 335-701 μmol/L (2.5th; 97.5th percentile; for 10-20 and 30-37 weeks gestation, respectively) for maternal plasma sulfate, and 0.06-0.31 and 0.05-0.28 for maternal FEI sulfate. Term venous cord plasma sulfate median levels were significantly (p = 0.038) higher in female babies (375 μmol/L) when compared to male babies (342 μmol/L), with an overall reference interval of 175-603 μmol/L.

**Conclusions:**

We provide the first reference intervals for maternal plasma sulfate levels and FEI sulfate, as well as cord plasma sulfate levels. These findings provide reference data for further studies of sulfate levels in both mother and child.

**Electronic supplementary material:**

The online version of this article (doi:10.1186/s12884-015-0526-z) contains supplementary material, which is available to authorized users.

## Background

Nutrient sulfate is the fourth most abundant anion in human circulation (approximately 300 μmol/L) and has numerous roles in human physiology [1,2]. Sulfate conjugation (sulfonation) of glycosaminoglycans such as cerebroside sulfate and heparan sulfate, contributes to the normal structure and function of tissues [3,4]. Sulfonation also detoxifies xenobiotics and certain pharmacological drugs such as acetaminophen [5,6], and in most cases inactivates steroids and iodothyronines [7-9]. In addition, sulfate transforms the biological activity of bile acids and catecholamines [10,11].

More than 20 genes involved in maintaining the required biological ratio of sulfonated and unconjugated molecules have been linked to pathophysiologies in humans and animals [12]. For example, genes encoding sulfatases, which mediate the removal of sulfate from proteoglycans or lipids, are linked to several lysosomal storage diseases, including metachromatic leukodystrophy, Maroteaux-Lamy syndrome, Morquio A syndrome, Sanfilippo A and D syndromes and Hunter syndrome [13]. In addition, the *SLC26A2* gene which mediates sulfate transport into chondrocytes for the sulfonation of chondroitin proteoglycan, is linked to four types of chondrodysplasias: multiple epiphyseal dysplasia (MEM), diastrophic dysplasia (DTD), atelosteogenesis Type II (AO2) and achondrogenesis Type IB (ACG1B) [14].

A sufficient supply of sulfate is required for maintaining intracellular sulfonation capacity. In adults and children, a well-balanced diet contributes approximately one third of body sulfate requirements (2.1-15.8 mmol/day) [15-18]. The remaining two thirds of sulfate requirements are derived from the intra-cellular metabolism of thiol compounds and the sulfur-containing amino acids methionine and cysteine [19,20].

The developing human fetus has negligible capacity to generate its own sulfate requirements, and thereby is reliant on sulfate supply from the maternal circulation via the placenta [21]. Sulfate transport through the placenta is mediated by the SLC13A4 sulfate transporter, which is abundantly expressed in the syncytiotrophoblast layer of the human and mouse placentae [22,23]. A related sulfate transporter, SLC13A1, is expressed in the maternal kidneys where it mediates sulfate reabsorption and maintains circulating sulfate levels [24].

During mouse gestation, increased *Slc13a1* gene expression in the maternal kidneys leads to increased renal sulfate reabsorption and a two-fold increase in circulating sulfate levels in the pregnant mouse [25]. The increased sulfate level in pregnant female mice is proposed to provide a reservoir of sulfate for supplying the high sulfate demands in the developing fetal tissues [12]. Targeted disruption of the *Slc13a1* gene in pregnant female mice leads to maternal renal sulfate wasting and hyposulfatemia, as well as fetal hyposulfatemia and mid-gestational miscarriage [25]. More recently, we have shown that loss of function mutations in the human *SLC13A1* gene lead to renal sulfate wasting and hyposulfatemia [26]. In addition, certain physiological conditions (vitamin D depletion, hypokalaemia, metabolic acidosis) and pharmacological drugs (NSAIDS, glucocorticoids) are known to down-regulate *SLC13A1* mRNA and protein expression [27]. Furthermore, dietary sulfate intake is correlated to circulating sulfate levels in both humans and rodents [28,29]. Collectively, these studies highlight the genetic, physiological and dietary contributions to modulation of circulating sulfate levels, which have potential clinical relevance to fetal growth and development in human gestation.

Despite its diverse and important roles in human physiology, sulfate is not routinely measured in clinical settings. Previous research studies have measured sulfate levels in biological fluids from relatively small cohorts using a range of methodologies, including barium-based turbidimetric assays, spectrophotometric benzidine precipitation methods and anion chromatography [1]. However, the clinical utility of those approaches for measuring sulfate have not been validated and therefore reference intervals for plasma sulfate and urinary sulfate excretion are not available. Interestingly, some research studies have indicated an increased sulfatemia in pregnant women when compared to non-pregnant women [30]. These earlier human studies, together with more recent animal research linking maternal hyposulfatemia in pregnancy with mid-gestational miscarriage [24,25], led us to establish validated tests for measuring sulfate in human plasma and urine in clinical settings [26], and to further investigate sulfate levels in human gestation.

In this paper we report reference intervals for maternal plasma sulfate levels and FEI sulfate in early and late human gestation, as well as venous cord plasma sulfate levels in term infants. Our data also considers the potential association of sulfate parameters with several prenatal factors, including maternal age, gestational age at recruitment and birth, gravidity, parity, vitamin supplements and gender of the fetus.

## Methods

The research protocol was approved by the Mater Health Services Human Research Ethics Committee. All women gave their written informed consent prior to their inclusion in the study. The eligibility criteria for our study were pregnant women ≥18 years of age who were attending for routine antenatal care at the Mater Mothers’ Hospital. Exclusion criteria were women with one or more of the following: maternal diabetes, hypertensive disorders, substance use, multi-fetal gestation, major congenital abnormality and fetal death. Gestational age at sample collection was based on best estimate, using menstrual dates corroborated by ultrasound or early gestation ultrasound. Maternal blood and urine sampling was aligned with routine clinical assessment visits within the antenatal clinic. Each spot urine sample (no preservative added) was collected at approximately the same time as a blood sample into a lithium heparin plasma separator tube (PST). Venous cord blood sampling (in K_2_EDTA) was aligned with routine collection from healthy term (37-41 weeks) deliveries (altruistic cord blood donors) at the Queensland Cord Blood Bank At The Mater.

Plasma and urinary sulfate were measured by ion chromatography with suppressed conductivity detection using a Dionex ICS2000, as previously described [26]. The coefficient of variation for sulfate analysis is <5 % for urine and < 7.5 % for plasma. Creatinine levels were quantitated using a Vitros 5.1 FS chemistry analyzer. Fractional excretion index (FEI) of sulfate was calculated using the formula (plasma creatinine [μmol/L] x urine sulfate [μmol/L]) / (urine creatinine [μmol/L] x plasma sulfate [μmol/L]). All samples were analysed blinded to operator.

The methodology for determination of the reference intervals for plasma sulfate and FEI sulfate has been based on the recommendations from the International Federation of Clinical Chemistry [31]. All primary reference ranges have been calculated using more than 40 samples, allowing for reliable estimates of the 2.5th and 97.5th centiles. A non-parametric calculation has been used for calculation of ranges due to non-normally distributed data, and the difficulty in interpreting log-transformed values.

A two-way Kruskal Wallis analysis of variance has been used to determine if there are significant differences in maternal plasma sulfate and FEI sulfate between both gender of the baby (males versus females) and gestational age at recruitment (10-20 weeks versus 30-37 weeks). Further analyses (Mann-Whitney U test) have been carried out to determine if within each gestational time period (10-20 weeks and 30-37 weeks) plasma sulfate and FEI sulfate differs for gender of the baby.

To determine if there are significant differences in maternal plasma sulfate and FEI sulfate within each gestational age (10-20 weeks and 30-37 weeks) with selected clinical parameters, various statistical approaches have been employed. Spearman’s correlation coefficient was used for gestational age at recruitment, gestational age at birth, birth weight, birth weight z-score (adjusted for gestational age and gender [32]), parity (grouped to 0, 1, 2, 3+) and gravidity (grouped to 0, 1, 2, 3, 4, 5+) while a two-way Kruskal-Wallis test was used for prenatal vitamin supplements that contain ferrous sulfate. In addition, Spearman’s correlation coefficients were used to determine if there are significant differences in cord plasma sulfate levels with maternal age and gestational age at birth.

All analyses have been carried out in StataSE version 10.1 (StataCorp Pty Ltd, College Station, Texas), except for two-way Kruskal Wallis that was performed in SPSS version 15 (IBM Corporation, Armonk, New York). Statistical significance is based on a two-tailed level of 0.05.

## Results

In this study, we recruited two cohorts of 120 pregnant women in early (10-20 weeks) and late (30-37 weeks) gestation. The sample sizes were reduced to 103 and 106, respectively, following exclusion criteria or withdrawal of consent that occurred post recruitment. For FEI sulfate calculations, the lack of urine sampling further reduced the sample sizes to 101 (early gestation) and 105 (late gestation). For cord plasma analyses, a sample size of 80 was reached following exclusion criteria or insufficient plasma volume for sulfate measurements. A summary of participant numbers in each sampling group stratified according to gender of baby, gestational age at birth, gravidity and parity, is shown in Table 1.

**Table 1:** **Summary of participant numbers from the two maternal gestational age groups and the cord plasma group stratified according to gender of baby, gestational age at birth, gravity and parity**

		**Maternal plasma and urine**				**Cord plasma**	
***Gestational age at sampling***		**10-20 wk**		**30-37wk**		**37-41 wk**	
***Gender of baby***		***Male***	***Female***	***Male***	***Female***	***Male***	***Female***
***Gestational age at birth*** ^***a***^							
30wk + 0d	36wk + 6d	2	2	7	3	-	-
37wk + 0d	37wk + 6d	6	3	4	4	4	0
38wk + 0d	38wk + 6d	10	12	8	10	17	26
39wk + 0d	39wk + 6d	13	16	15	10	9	14
40wk + 0d	43wk + 6d	21	18	24	21	4	6
***Gravidity*** ^***b***^	G1	21	19	13	11	-	-
	G2	17	11	20	10	-	-
	G3	4	9	12	11	-	-
	G4	4	5	5	9	-	-
	G5+	6	7	8	7	-	-
***Parity*** ^***b***^	P0	28	23	20	16	-	-
	P1	11	13	23	18	-	-
	P2	7	10	9	9	-	-
	P3+	6	5	6	5	-	-

^a^The gestational age at birth is shown as a range of weeks (wk) and days (d).

^b^Gravidity (G) and parity (P) was obtained only from participants in the 2 cohorts contributing maternal plasma and urine samples at 10-20 and 30-37 weeks gestation.

### Maternal plasma sulfate levels and FEI sulfate in pregnancy

**Figure 1:**
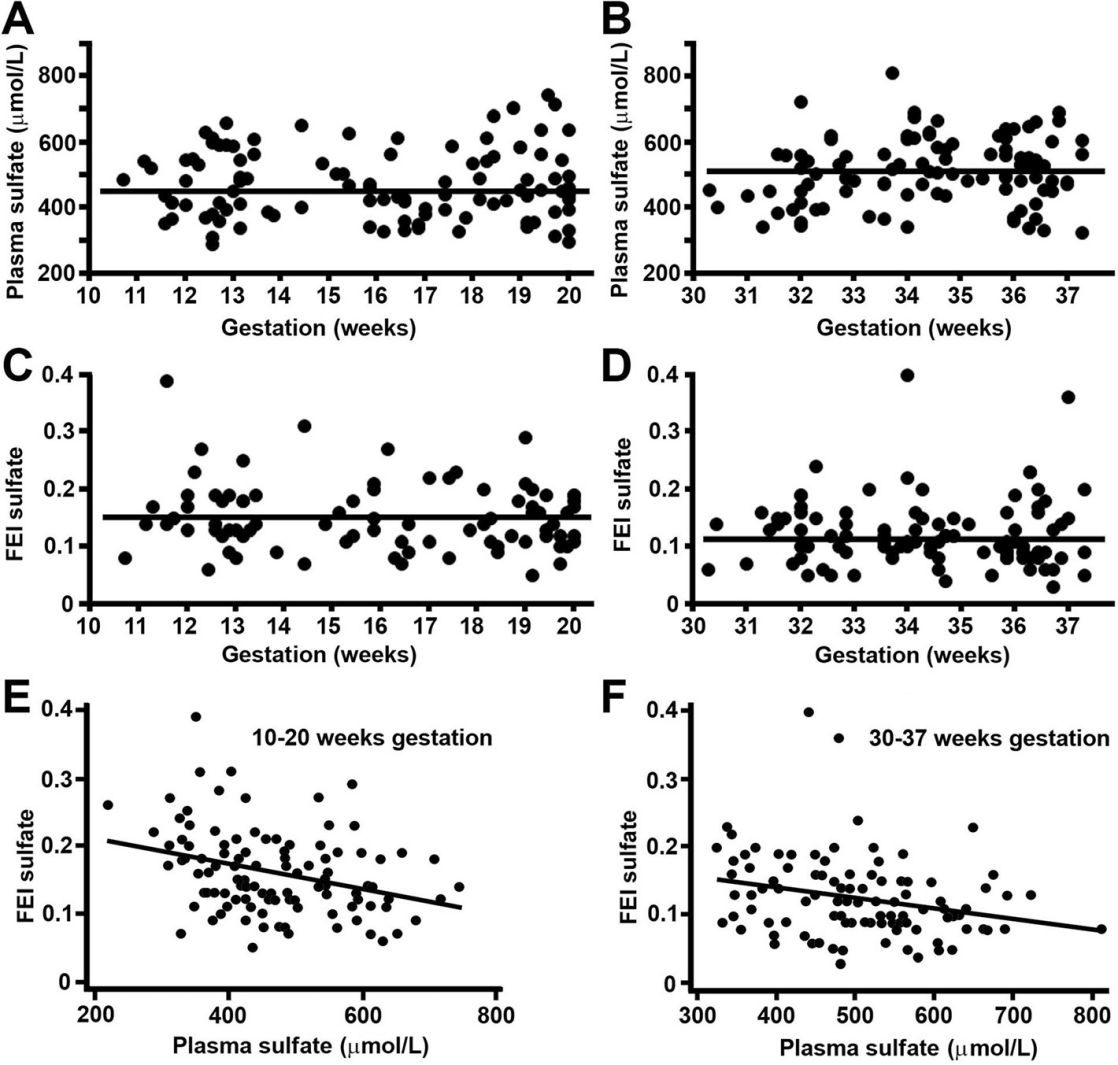
Plasma sulfate and FEI sulfate levels in pregnant women. **(A-B)** Plasma sulfate levels and **(C-D)** FEI sulfate levels in **(A, C)** early (10-20 weeks) and **(B, D)** late (30-37 weeks) gestation. Individual data and median (*bar*). **(E-F)** Maternal plasma sulfate levels inversely correlate to FEI sulfate. Individual data and trend line for **(E)** early gestation, rho = -0.335, p < 0.001 and **(F)** late gestation, rho = -0.281, p = 0.004.

The 95% reference intervals for maternal plasma sulfate and FEI sulfate are shown in Table 2. Median plasma sulfate levels were significantly increased at 30-37 weeks gestation (502 μmol/L, p = 0.006) when compared to levels at 10-20 weeks gestation (452 μmol/L) (Figure 1A-B, Tables 3 and 4). Maternal plasma sulfate levels were similar when carrying a male or female fetus (Tables 3 and 4).

**Table 2:** **Reference intervals for maternal and cord plasma sulfate parameters**

**Gender baby**	**N**	**Lower bound** ^**a**^ **(90% CI)** ^**b**^	**Upper bound** ^**a**^ **(90% CI)** ^**b**^
*Maternal plasma sulfate (μmol/L) 10-20 weeks gestation*			
All	103	305 (220, 327)	710 (651, 743)
Male	52	244 (220, 313)	695 (615, 716)
Female	51	316 (310, 336)	732 (661, 743)
*Maternal plasma sulfate (μmol/L) 30-37 weeks gestation*			
All	106	335 (325, 344)	701 (667, 811)
Male	58	328 (325, 367)	699 (637, 721)
Female	48	338 (337, 344)	784 (672, 811)
*Maternal FEI sulfate 10-20 weeks gestation*			
All	101	0.06 (0.05, 0.07)	0.31 (0.27, 0.39)
Male	51	0.06 (0.06, 0.07)	0.30 (0.26, 0.31)
Female	50	0.06 (0.05, 0.09)	0.37 (0.27, 0.39)
*Maternal FEI sulfate 30-37 weeks gestation*			
All	105	0.05 (0.03, 0.05)	0.28 (0.22, 0.40)
Male	58	0.04 (0.03, 0.06)	0.38 (0.20, 0.40)
Female	47	0.04 (0.04, 0.05)	0.23 (0.20, 0.23)
*Cord plasma sulfate (μmol/L) 37-41 weeks gestation*			
All	80	175 (159, 216)	603 (553, 683)
Male	34	159 (159, 216)	598 (505, 598)
Female	46	186 (180, 243)	669 (538, 683)

N: Sample size.

^a^Lower and Upper bounds represent the 95% reference intervals.

^b^90% confidence intervals around each lower and upper bound are shown in parentheses.

**Table 3:** **Descriptive statistics for maternal and cord plasma sulfate parameters**

**Gender baby**	**N**	**Range**	**Median**	**IQR (25** ^**th**^ **–75** ^**th**^ **)**
All	103	220-743	452	166 (381-547)
Male	52	220-716	457	173 (364-537)
Female	51	310-743	452	183 (403-586)
*Maternal plasma sulfate (μmol/L) 30-37 weeks gestation*				
All	106	325-811	502	126 (439-565)
Male	58	325-721	490	101 (449-550)
Female	48	337-811	533	198 (407-605)
*Maternal FEI sulfate 10-20 weeks gestation*				
All	101	0.05-0.39	0.15	0.08 (0.12-0.20)
Male	51	0.06-0.31	0.14	0.09 (0.11-0.20)
Female	50	0.05-0.39	0.16	0.09 (0.12-0.21)
*Maternal FEI sulfate 30-37 weeks gestation*				
All	105	0.03-0.40	0.11	0.06 (0.09-0.15)
Male	58	0.03-0.40	0.13	0.07 (0.09-0.16)
Female	47	0.04-0.23	0.10	0.06 (0.08-0.14)
*Cord plasma sulfate (μmol/L) 37-41 weeks gestation*				
All	80	159-683	357	131 (292-423)
Male	34	159-258	342	133 (258-391)
Female	46	180-683	375	128 (322-450)

N: Sample size; IQR: interquartile range, with lower (first) and upper (third) quartile shown in parentheses.

**Table 4:** **Comparison of plasma sulfate levels and FEI sulfate between gender and gestational age at recruitment**

**Parameter**	**Factor**	*p-Value
Plasma sulfate	Gender	-
	Time (10-20 versus 30-37 weeks)	0.006
	Interaction	-
FEI sulfate	Gender	-
	Time (10-20 versus 30-37 weeks)	<0.001
	Interaction	0.038

Comparison of gender, gestational age of maternal plasma and urine sampling (Time), and both Gender and Time (Interaction) for plasma sulfate levels and FEI sulfate. FEI: Fractional Excretion index. *Only values <0.05 are shown.

Median FEI sulfate values were significantly lower at 30-37 weeks gestation (0.11, p < 0.001) when compared to values at 10-20 weeks gestation (0.15) (Figure 1C-D, Tables 3 and 4), and were inversely correlated to plasma sulfate levels at 10-20 weeks gestation (rho = -0.335, p < 0.001) and 30-37 weeks gestation (rho = -0.281, p = 0.004), respectively (Figures 1E-F). There was a significant interaction of gender and FEI sulfate when comparing 10-20 and 30-37 weeks gestation (p = 0.038) (Table 4, Additional file 1: Figure S1), indicating a marked decline in maternal FEI sulfate as pregnancy progressed when the fetus is female.

### Investigation of maternal plasma and urinary sulfate data and prenatal factors

We performed analyses on the maternal plasma sulfate and FEI sulfate data with several prenatal factors (Table 5). No significant association was found for gestation at recruitment or birth, birth weight, birth weight z-score and prenatal multi-vitamin supplements. Maternal FEI sulfate values at 30-37 weeks gestation correlated negatively with gravidity (rho = -0.289, p = 0.003) and parity (rho = -0.281, p = 0.004).

**Table 5:** **Analysis of maternal sulfate data and clinical parameters**

**Clinical parameter**	**Rho** ^**b**^	*p-Value
*Maternal plasma sulfate 10-20 weeks gestation*		
Gestational age at recruitment	0.004	-
Gestational age at birth	0.016	-
Birth weight	0.156	-
Birth weight z-score^a^	0.103	-
Gravidity	0.019	-
Parity	0.041	-
Vitamin supplement	-	-
*Maternal plasma sulfate 30-37 weeks gestation*		
Gestational age at recruitment	0.140	-
Gestational age at birth	0.147	-
Birth weight	-0.047	-
Birth weight z-score^a^	-0.126	-
Gravidity	0.097	-
Parity	0.151	-
Vitamin supplement	-	-
*Maternal FEI sulfate 10-20 weeks gestation*		
Gestational age at recruitment	-0.082	-
Gestational age at birth	0.012	-
Birth weight	-0.088	-
Birth weight z-score^a^	-0.043	
Gravidity	-0.094	-
Parity	-0.013	-
Vitamin supplement	-	-
*Maternal FEI sulfate 30-37 weeks gestation*		
Gestational age at recruitment	-0.037	-
Gestational age at birth	0.115	-
Birth weight	0.090	-
Birth weight z-score^a^	0.003	-
Gravidity	-0.289	0.003
Parity	-0.281	0.004
Vitamin supplement	-	-

^a^Adjusted for gestational age and gender.

^b^Rho: Spearman’s correlation coefficient.

*Only values <0.05 are shown.

### Term cord plasma sulfate levels

Reference intervals for term cord plasma sulfate are shown in Table 2. Analysis of 80 samples showed a median plasma sulfate level of 357 μmol/L (Table 3), with no significant correlation to maternal age (range 24-42 years) or gestational age (range 37-41 weeks) (Table 6). However, median plasma sulfate levels were significantly (p = 0.038) higher for female babies (375 μmol/L, n = 46) when compared to male babies (342 μmol/L, n = 34) (Tables 3 and 6).

**Table 6:** **Analysis of cord plasma sulfate data and clinical parameters**

**Clinical parameter**	**Rho** ^**a**^	*p-Value
*Cord plasma sulfate 37-41 weeks gestation*		
Maternal age	-0.010	-
Gestational age at birth	-0.107	-
Gender of baby	-	0.038

^a^Rho: Spearman’s correlation coefficient.

*Only values <0.05 are shown.

## Discussion

In this study, we report the first set of reference intervals for plasma sulfate and FEI sulfate using a validated sulfate test and provide new evidence on the physiological regulation of sulfate in pregnancy. Our data extend previous research findings that show human plasma sulfate levels are increased in early pregnancy compared to healthy males and non-pregnant females with peak values occurring at 30-37 weeks gestation [1,30]. In a previous study, we reported similar findings in mice [25]. In addition, we noted a negative correlation of FEI sulfate (i.e. increasing renal sulfate reabsorption) with gravidity and parity at 30-37 weeks gestation, with a steeper decline occurring in female babies when compared to male babies. Our data suggest that the gender of the fetus can influence sulfate levels in cord blood as well as reabsorption of sulfate in the maternal kidneys.

Several earlier studies using ion chromatography reported plasma sulfate median levels of approximately 300 μmol/L in adult males and non-pregnant females [1]. In the present study, we found an approximate 1.5-fold increase (452 μmol/L) at 10-20 weeks gestation when compared to published data on non-pregnant women, with median levels peaking (502 μmol/L) at 30-37 weeks gestation. This increased sulfatemia in pregnant women is remarkable and suggests active up-regulation of plasma sulfate levels, as many circulating analytes usually decrease slightly during gestation as a result of changes in extracellular fluid volume and renal function [33,34].

Similar increases in circulating sulfate levels have been reported for pregnant mice, with a two-fold increase and levels peaking in late gestation [25]. The increased sulfatemia in pregnant mice is due to increased expression of the renal Slc13a1 sulfate transporter, which mediates sulfate reabsorption in the maternal kidneys [25]. Disruption of the *Slc13a1* gene in pregnant mice leads to maternal and fetal hyposulfatemia, as well as late gestational miscarriage [25]. These findings highlight the importance of Slc13a1 for maintaining high maternal sulfatemia, which supplies the high fetal demands for sulfate throughout pregnancy.

We have linked loss of function mutations (R12X and N174S) in the human *SLC13A1* gene with renal sulfate wasting and hyposulfatemia [26]. The nonsense R12X variant led to complete loss of SLC13A1 function and has an allelic frequency of 0.36% in the general population, whereas the N174S variant led to partial loss of function (60% decrease) and has an allelic frequency of 26.99% [35]. Genetic screening for these two variants was not performed in the current study. However, the relatively high frequency of N174S may be relevant to the varying plasma sulfate levels and FEI sulfate found in our two cohorts of pregnant women. Nonetheless, our findings of increasing sulfatemia in pregnancy is most likely due to enhanced SLC13A1-mediated sulfate reabsorption in the maternal kidneys, as suggested by the negative correlation between maternal plasma sulfate levels and fractional urinary sulfate excretion.

Of great interest is the slightly higher cord venous plasma sulfate level in female babies when compared to males. Cord venous blood flows from the placenta to the fetus and therefore its composition reflects the supply of analytes derived from the maternal circulation. Accordingly, our data reflects a higher net sulfate transport through the maternal-fetal barrier for female babies when compared to males. The SLC13A4 sulfate transporter is localised to the syncytiotrophoblast layer of human placenta where it is proposed to be mediating sulfate supply from mother to fetus [23]. Male and female babies have similar placental SLC13A4 levels [23], suggesting that SLC13A4 is unlikely to be the cause of higher plasma sulfate levels in female babies. In a previous study, we reported that another placental sulfate transporter, SLC26A2, is more abundant in cytotrophoblasts of male babies when compared to female babies [23]. This gender difference for placental SLC26A2 expression suggests a higher sulfate requirement in cytotrophoblasts of male babies, which may potentially limit sulfate supply to the male fetus. In addition, the present study also shows a more rapid decline in maternal FEI sulfate (i.e. increasing renal sulfate reabsorption) during pregnancy when the fetus is female. This latter finding may suggest a maternal contribution to the increased cord plasma sulfate levels for female babies.

Interestingly, we show a negative correlation for FEI sulfate with gravidity and parity in the cohort of women at 30-37 weeks gestation. This finding suggests that increased sulfate reabsorption in late gestation (i.e. lower FEI sulfate values) is more prevalent for those women with the highest number of previous pregnancies and births. This finding may be relevant to our animal studies that showed increased renal sulfate reabsorption in late gestation is important for maintaining normal pregnancy and high fecundity [25]. Since the present study excluded data from women when major congenital abnormality and fetal death occurred, we are unable to determine whether high FEI sulfate and low plasma sulfate levels lead to perturbed fetal development and/or fetal loss. However, that will be the next phase of our work.

Further studies are warranted to explore the physiological roles of sulfate in pregnancy; the differences observed in FEI sulfate levels at 30-37 weeks gestation in mothers with increasing gravidity and parity and female infants; the gender differences noted in cord plasma sulfate levels; and whether low maternal sulfate levels lead to perturbed fetal development.

## Conclusions

We provide the first set of reference intervals for maternal plasma sulfate and FEI sulfate in early and late gestation, and for term venous cord plasma sulfate, using a validated sulfate test. Our findings show increased maternal plasma sulfate levels in pregnancy with levels peaking within 30-37 weeks gestation. This increased sulfatemia is inversely correlated to fractional urinary sulfate excretion, indicating the contribution of the maternal kidneys to maintaining high circulating sulfate levels in human gestation. Our data also show higher term venous cord plasma sulfate levels for female babies when compared to male babies. Collectively, this study provides reference data for future clinical studies of sulfate levels in human gestation, and warrants investigation into the consequences of abnormal plasma sulfate levels in mother and child.

## Additional file

Additional file 1: Figure S1. Relationship between median maternal FEI sulfate, gender of fetus and gestational age. A significant interaction (p=0.038) between gender suggests that as gestation progresses, maternal renal sulfate reabsorption is higher when carrying a female fetus.

## Abbreviations

FEI: Fractional Excretion Index
MEM: Multiple epiphyseal dysplasia
DTD: Diastrophic dysplasia
AO2: Atelosteogenesis Type II
ACG1B: Achondrogenesis Type IB
NSAIDS: Nonsteroidal anti-inflammatory drugs
IQR: Interquartile range
