# Supplementary material for: Reference intervals for plasma sulfate and urinary sulfate excretion in pregnancy
Source: BMC Pregnancy Childbirth. 2015 Apr 17;15:96. doi: 10.1186/s12884-015-0526-z (PMC4404267; doi:10.1186/s12884-015-0526-z)
Supplement: Additional file 1: Figure S1. — Relationship between median maternal FEI sulfate, gender of fetus and gestational age. A significant interaction (p=0.038) between gender suggests that as gestation progresses, maternal renal sulfate reabsorption is higher when carrying a female fetus. [file 12884_2015_526_MOESM1_ESM.pdf]

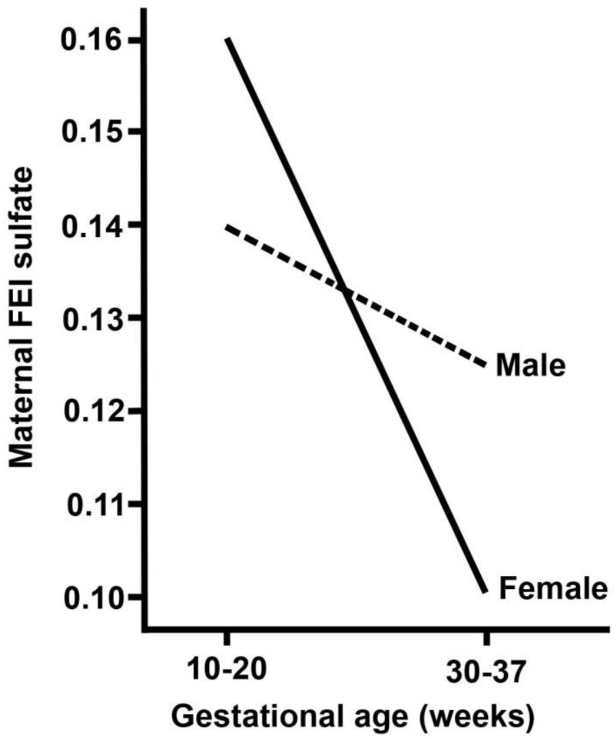

Supplemental Data Figure 1. Relationship between median maternal FEI sulfate, gender of fetus and gestational age. A significant interaction (p=0.038) between gender suggests that as gestation progresses, maternal renal sulfate reabsorption is higher when carrying a female fetus.
